# Reply to Meule, A. Comment on “Calugi et al. The Role of Weight Suppression in Intensive Enhanced Cognitive Behavioral Therapy for Adolescents with Anorexia Nervosa: A Longitudinal Study. *Int. J. Environ. Res. Public Health* 2023, *20*, 3221”

**DOI:** 10.3390/ijerph20176691

**Published:** 2023-08-31

**Authors:** Simona Calugi, Anna Dalle Grave, Maddalena Conti, Laura Dametti, Mirko Chimini, Riccardo Dalle Grave

**Affiliations:** Department of Eating and Weight Disorders, Villa Garda Hospital, Via Monte Baldo 89, 37016 Verona, Italy; annadallegrave@gmail.com (A.D.G.); missmadda@hotmail.com (M.C.); lauradametti96@gmail.com (L.D.); mirko.chimini1@gmail.com (M.C.); rdalleg@gmail.com (R.D.G.)

We read the comments by Meule on our article with great interest, and we thank the author for his thoughtful suggestions [1]. Meule commented on our data suggesting including z-body mass index (BMI) in the regression analysis despite the high multicollinearity to assess the role of this variable in weight change. We have two considerations to make regarding this comment. Firstly, we take the occasion to specify further that our study aimed not to evaluate body weight change but body weight at the end of treatment (and at follow-up), which is considered a predictor of long-term outcome [2]. Secondly, the suggestion of not worrying about multicollinearity is questionable. Indeed, if the degree of correlation between variables is high enough, it can cause problems fitting the model and interpreting the results.

Anyway, following Meule’s suggestion, we re-run the linear regression analysis, including baseline z-BMI, and found that developmental weight suppression (DWS) is positively correlated with end-of-treatment (EOT) z-BMI (beta = 0.60, t = 2.47, *p* = 0.015) but not with 20-week follow-up z-BMI (beta = 0.55, t = 1.79, *p* = 0.078). However, it should be noted that although the presence of multicollinearity does not reduce the predictive power or the reliability of the statistical model, the interpretation of the results of each individual predictor remains problematic [3,4]. As DWS was calculated as the difference between the highest premorbid z-BMI and the current z-BMI in our study, it is strictly influenced by the current z-BMI, with a negative correlation of −0.92. For this reason, it is unsurprising that adding current z-BMI in the statistical model interferes with its interpretation and could lead to misleading conclusions.

Meule also suggested evaluating the role of DWS without the covariates in the regression model. We found that in the logistic analysis, DWS was negatively associated with “good BMI outcome” and “full response” at EOT (Wald test = 7.83, *p* = 0.005; Wald test = 4.11, *p* = 0.043; respectively). However, as reported in our paper, this relationship disappears when including the covariates. Similarly, the linear regression analysis indicated that DWS was negatively correlated with EOT and the 20-week follow-up z-BMIs (beta = −0.41, t = −4.58, *p* < 0.001; beta = −0.32, t = −3.00, *p* = 0.004, respectively).

We take the occasion to underline that the decision to include several covariates in the statistical model was based on clinical considerations. We hypothesized that BMI at the end of treatment and follow-up could be influenced by different variables, including age, illness duration, eating disorder and general psychopathology, and clinical impairment, and we aimed to evaluate the incremental role of DWS in the statistical model.

We think that our study could provide new stimuli for reflection by trying to break down preconceived ideas.

## Data Availability

The data presented in this study are available on request from the corresponding author. The data are not publicly available due to privacy issues.
